# Functional mapping of the somatotopic organization of the supplementary motor area using navigated repetitive transcranial magnetic stimulation and computer vision-based analysis

**DOI:** 10.3389/fnins.2026.1698148

**Published:** 2026-01-30

**Authors:** Jonathan Stein, Thomas Picht, Melina Engelhardt

**Affiliations:** 1 Charité – Universitätsmedizin Berlin, Corporate Member of Freie Universität Berlin and Humboldt-Universität zu Berlin, Department of Neurosurgery, Charitéplatz 1, Berlin, Germany; 2 Charité – Universitätsmedizin Berlin, Corporate Member of Freie Universität Berlin and Humboldt-Universität zu Berlin, Einstein Center for Neurosciences, Charitéplatz 1, Berlin, Germany; 3 Cluster of Excellence Matters of Activity, Image Space Material, Humboldt-Universität zu Berlin, Berlin, Germany; 4 Department of Neuroscience and Biomedical Engineering, Aalto University School of Scienc, Espoo, Finland

**Keywords:** computer vision techniques, functional assessment, motor function, nrTMS, somatotopy, supplementary motor area

## Abstract

**Background:**

The supplementary motor area (SMA) is a cortical region involved in motor and language functions. Motor representations within the SMA follow a somatotopic organization: Anterior regions are linked to orofacial movements, middle regions to upper limb movements, and posterior regions to lower limb movements. SMA lesions may produce impairments that correspond to this somatotopy; therefore, preoperative assessment may aid diagnosis.

**Objective:**

This study aimed to revise and extend a protocol for assessing the SMA using navigated repetitive transcranial magnetic stimulation (nrTMS), incorporating somatotopic organization and validating positive stimulation points against non-motor regions.

**Methods:**

The dominant-hemisphere SMA of 30 healthy participants (27.1 ± 6.21 years, 18 female) was examined. After mapping of the primary motor cortex with single-pulse TMS, six predefined SMA sites were stimulated using 20 Hz nrTMS while participants performed the Nine Hole Peg Test (NHPT; 120% resting motor threshold (RMT)), the lower extremity motor coordination test (LEMOCOT; 140% RMT), and an orofacial task (130% RMT). Each test was repeated under identical parameters at non-motor control sites. Kinematic measurements were obtained using high-speed recordings.

**Results:**

SMA stimulation disrupted upper extremity function, with the strongest effects observed at posterior sites. In contrast, lower extremity performance was not impaired during SMA stimulation, where tapping speed increased under validation conditions. Orofacial effects were limited and inconsistent, occurring mainly during stimulation outside the SMA and showing no significant spatial pattern.

**Conclusion:**

The expected somatotopic organization of the SMA could not be demonstrated using nrTMS. However, SMA-selective disruptions of upper extremity movements suggest a functional, rather than effector-specific, organization. The novel kinematic paradigm enabled detailed, objective analysis of movement phases and may benefit future TMS studies.

## 1 Introduction

The supplementary motor area (SMA) is a cortical region located on the dorsomedial frontal cortex, anterior to the primary motor cortex (M1; Nachev et al., 2008; Picard and Strick, 2001).

Functionally, the SMA contributes to diverse cognitive domains, including speech and language (Hertrich et al., 2016), auditory processing (Lima et al., 2016), and spatial processing (Zacks, 2008), as well as to motor behaviors such as sequential movement control (Nachev et al., 2008; Ohbayashi, 2021; Tanji, 2001), inhibition of voluntary motor plans (Boy et al., 2010), and self-initiated movement generation (Passingham et al., 2010).

Damage to the SMA, for instance, observed after brain tumor resection, underscores its role in cognitive and motor functions. Such lesions may lead to supplementary motor area syndrome (SMAS), which presents with symptoms such as contralateral akinesia and mutism (Laplane et al., 1977; Palmisciano et al., 2022; Zentner et al., 1996). Symptom patterns vary with lesion location: Anterior left SMA damage typically causes language deficits, while anterior-to-posterior lesion progression may impair face, upper limb, and lower limb functions, suggesting a somatotopic organization (Fontaine et al., 2002). This organization has been observed in studies using subdural electrical stimulation (Fried et al., 1991; Hanakawa et al., 2001; Yazawa et al., 2000), subdural electrophysiology recordings (Ikeda et al., 1992), and fMRI (Chainay et al., 2004; Mayer et al., 2001; Zeharia et al., 2012).

Although SMAS is frequently transient in nature (Palmisciano et al., 2022; Pinson et al., 2021; Tsai et al., 2022), fine motor impairments can persist for several months in some patients (Maurer et al., 2024). In individuals with reduced life expectancy, the occurrence of SMAS imposes an additional clinical burden (Potgieser et al., 2014).

The extent of SMA resection is associated with the likelihood of postoperative neurological deficits (Zentner et al., 1996; Russell and Kelly, 2003) and represents a significant predictor of such outcomes (Kim et al., 2013).

Despite this clinical relevance, SMA mapping remains uncommon. Earlier studies have relied on task-based fMRI (Hiroshima et al., 2014; Nelson et al., 2002), which can be challenging to integrate into clinical workflows (Engelhardt et al., 2023). Alternative resting-state fMRI offers inconsistent localization and visibility (Kokkonen et al., 2009). Recently, navigated repetitive transcranial magnetic stimulation (nrTMS) has emerged as a promising tool, with studies showing that SMA stimulation disrupts motor performance in healthy individuals (Schramm et al., 2019, 2020). Following this, an nrTMS protocol was developed to map upper extremity function in the dominant hemisphere (Engelhardt et al., 2023) and was later expanded to include bilateral SMA and lower extremity functions (Kern et al., 2023).

However, these previous studies have not systematically assessed the full somatotopic organization of the SMA, omitting orofacial movements and lacking appropriate control conditions outside the SMA to validate effect specificity.

The current study addresses these gaps by extending nrTMS protocols to map anterior, middle, and posterior SMA subregions associated with orofacial, upper extremity, and lower extremity functions. It incorporates stimulation of non-motor control regions to assess the specificity of observed effects and introduces kinematic analysis using computer-vision techniques to enhance precision. This approach tests the hypothesis that the SMA is somatotopically organized along an anterior–posterior axis, with representations for the face, upper extremities, and lower extremities. It is expected that stimulation of specific SMA subregions will selectively impair the performance of the corresponding effector and that task disruption will occur during SMA stimulation but not during M1 stimulation or under non-stimulation conditions.

## 2 Methods

### 2.1 Participants

A total of 30 healthy participants (mean age 27.1 ± 6.21 years; 18 female; 27 right-handed) took part in the study. None of the participants met any of the exclusion criteria, which included psychological or neurological illness, migraines, tinnitus, limb or facial paresis, epilepsy or seizures (personal or familial), pregnancy, prescription medication use within the last 14 days, contraindications for MRI as per the Berlin Center for Advanced Neuroimaging (BCAN) guidelines, and contraindications for TMS (Rossi et al., 2021). The current study received approval from the Ethics Committee of Charité Universitätsmedizin Berlin and was conducted in accordance with the Declaration of Helsinki. All participants provided written informed consent after being fully informed about the study.

### 2.2 Magnetic resonance imaging

Participants who had not previously undergone an MRI received a T1-weighted MPRAGE sequence (TR = 2.530 ms, TE = 4.94 ms, TI = 1.100 ms, flip angle = 7, voxel size = 1 mm x 1 mm x 1 mm, 176 slices) on a Siemens 3-T Magnetom Trio MRI scanner (Siemens AG, Erlangen, Germany) at the BCAN.

### 2.3 Navigated transcranial magnetic stimulation

A navigated TMS system (NBS 5; Nexstim, Helsinki, Finland) with a 70 mm biphasic figure-of-eight coil was employed. For navigation, the participant’s MRI served as a reference. The TMS assessment was divided into two main parts. First, using single-pulse navigated TMS (nTMS), M1 of the dominant hemisphere was examined. Subsequently, the SMA was mapped using nrTMS.

#### 2.3.1 Mapping of the primary motor cortex

M1 mapping was conducted by recording motor evoked potentials (MEPs) from the first dorsal interosseous (FDI) muscle of the dominant hand using Ag/AgCl surface electrodes (Neuroline 720; Ambu, Ballerup, Denmark) in a belly–tendon configuration, with the ground electrode placed on the left palmar wrist. A standard operating protocol for M1 mapping was performed: Participants relaxed their hands, maintaining activation below 10 μV. nTMS was applied to M1 to identify the stimulation site, angulation, and electric field direction within M1 producing the largest MEPs in the FDI. The resting motor threshold (RMT) at this location was subsequently estimated using the system’s algorithm (Engelhardt et al., 2019). Using nTMS at 105% of the RMT, the cortical representation of the target muscle was identified (Engelhardt and Picht, 2020). To ensure that subsequent SMA mapping was not confounded by direct activation of M1, the area obtained through motor mapping was used to delineate M1 from the SMA. Although direct stimulation of M1 was thus avoided, posterior SMA stimulation may have induced an electric field exceeding the activation threshold in M1 due to the close proximity of these regions.

#### 2.3.2 Functional mapping of the somatotopic organization of the supplementary motor area

Following Engelhardt et al. (2023), the SMA was delineated (Vorobiev et al., 1998) as the superior frontal gyrus section anterior to M1, intersected by the vertical commissure anterior, using structural MRI. This region was evaluated for its functional representations of orofacial, upper extremity, and lower extremity movements by systematically applying stimulation across the SMA and observing consequent disruptions.

To this end, three motor tests were used to assess orofacial, upper extremity, and lower extremity movements as part of the mapping protocol. Each modality was assessed separately, and the order in which the modalities were tested was counterbalanced.

For the upper extremity movements, an adapted Nine Hole Peg Test (NHPT; Johansson and Häger, 2019) was used, given its established sensitivity to SMA-related impairment (Kern et al., 2023; Schramm et al., 2019). In this test, participants were required to unimanually place six pegs into a pegboard following a start signal. Lower extremity function was tested using the lower extremity motor coordination test (LEMOCOT; Desrosiers et al., 2005), where, after a start signal, participants were instructed to tap between two circular targets with their foot. Due to the lack of appropriate tests for orofacial movements, a novel motor task (FACE) was developed, drawing on video-based facial palsy assessments (Schaede et al., 2017) and EMG studies of facial muscle recruitment (Mueller et al., 2022; Schumann et al., 2010, 2021). The six movements included eye closure, lip puckering, eyebrow raising with forehead wrinkling, mouth-corner depression, cheek puffing, and closed-mouth smiling, representing distinct facial action units according to the Facial Action Coding System (Ekman and Friesen, 1978). Participants were shown reference images with instructions and asked to reproduce each movement.

**Figure 1:**
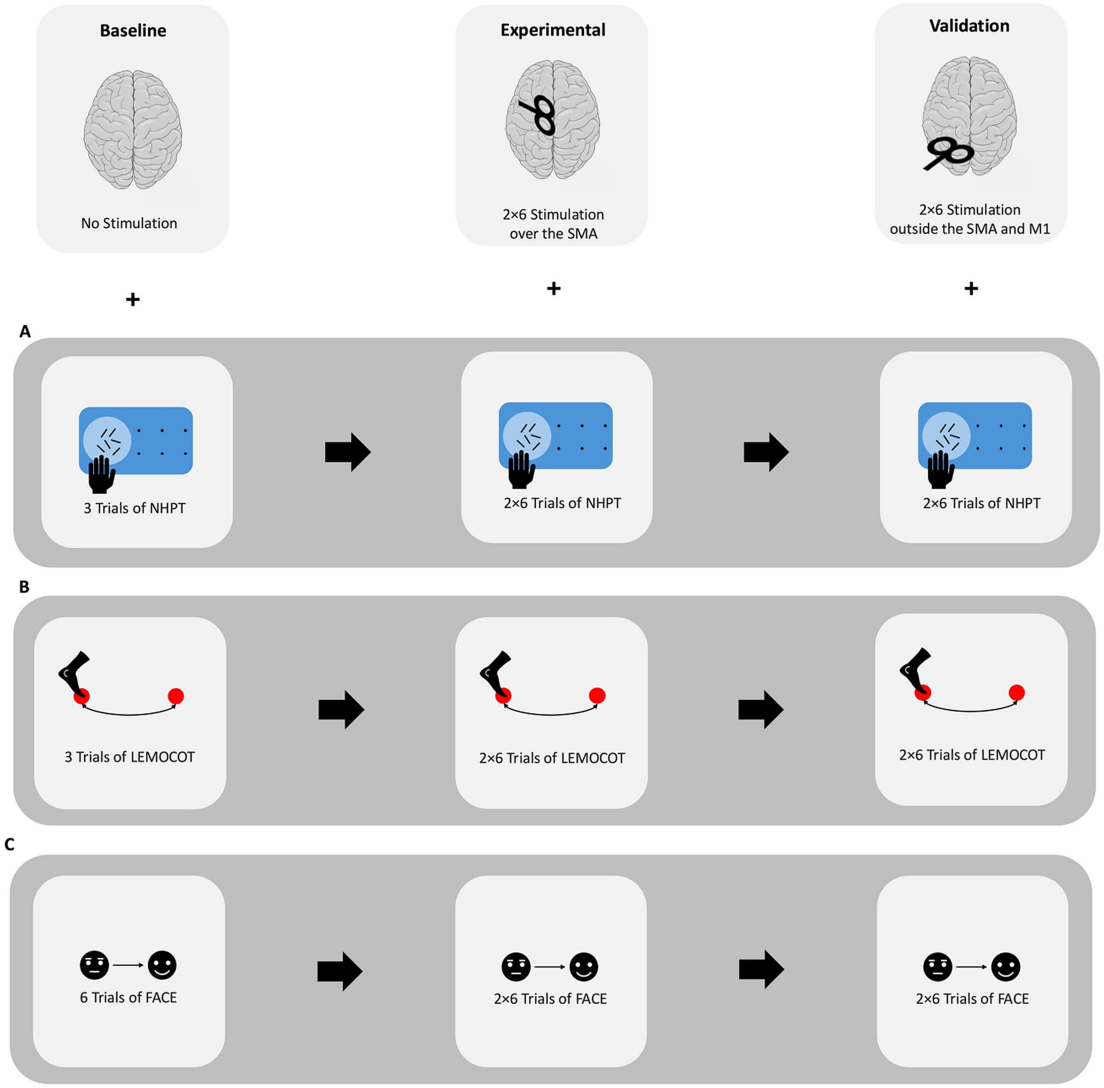
Overview of each mapping procedure, categorized by extremity. Testing was performed sequentially for the upper extremity **(A)**, lower extremity **(B)**, and face **(C)**. The order in which these extremities were tested was counterbalanced.

The mapping protocol was divided into three conditions: Baseline, experimental, and validation (Figure 1).

Participants practiced each task once to ensure comprehension. All stimuli were shown on a 27-inch screen using the NexSpeech Software (Nexstim, Helsinki, Finland).

Under the baseline condition, participants performed each motor task without stimulation. For orofacial movements, each of the six facial stimuli was displayed for 5 s, during which participants executed, briefly held, and then released the expression, followed by a 20-s inter-trial interval (ITI). For the upper and lower extremities, adapted versions of the NHPT and LEMOCOT were each performed three times for a maximum of 10 s, with a 40-s ITI. Trial onset and offset were cued using the system’s integrated display.

**Figure 2:**
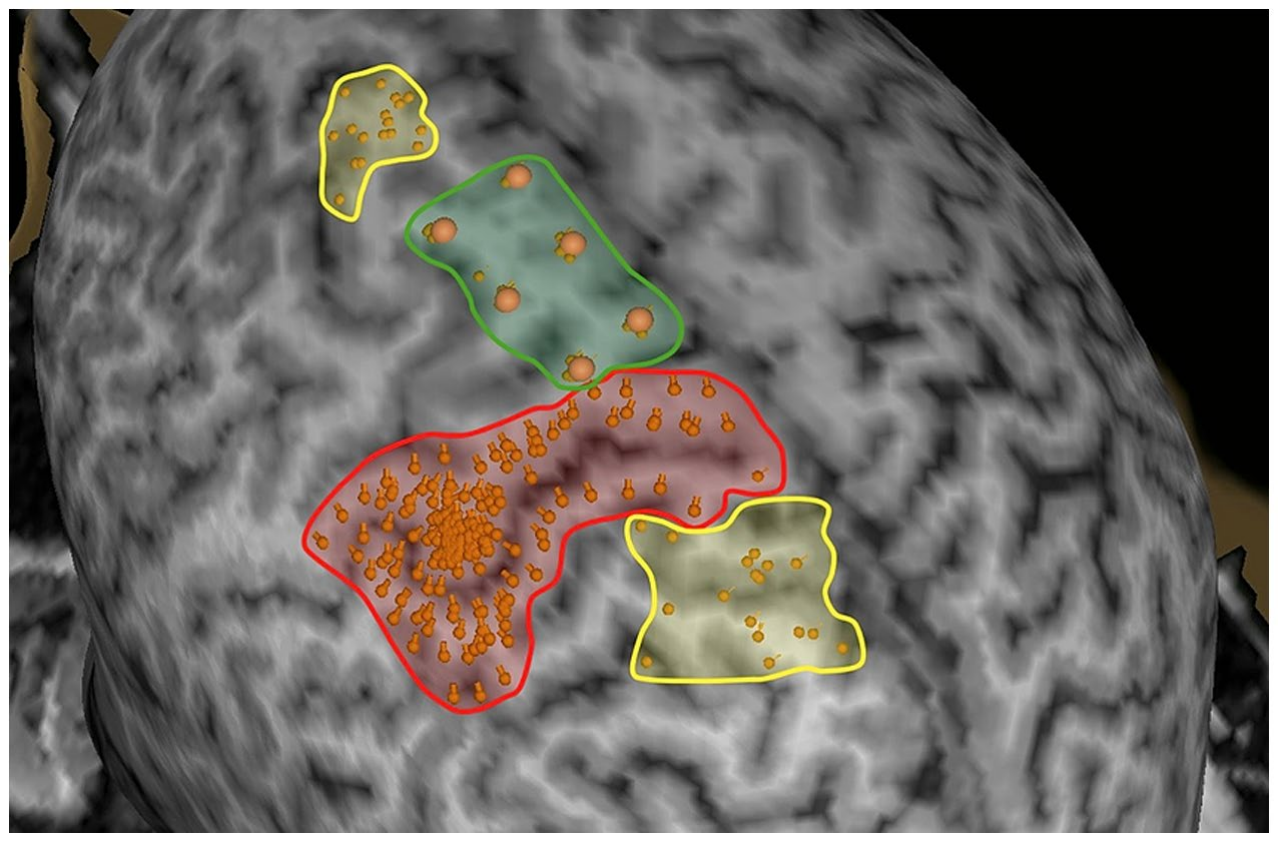
Example stimulation points for M1, SMA, and validation mapping. Points within the red region delineate the primary motor cortex, as estimated by single-pulse TMS. The green region indicates SMA stimulation targets arranged in a 2 × 3 array. Points within the yellow regions represent validation stimulation locations outside M1 and the SMA. The validation points shown here are exemplary, and validation stimulation was applied to varying locations beyond those displayed.

Under the experimental condition, nrTMS was applied to six manually selected targets within the SMA, arranged in a 2 × 3 array (Figure 2).

For orofacial mapping, participants performed each facial movement twice while receiving nrTMS bursts to the stimulation targets (20 Hz, 130% RMT, 5 s duration, ITI 20s), with the coil positioned perpendicular to the interhemispheric cleft. For upper extremity and lower extremity mapping, participants performed the NHPT and LEMOCOT 12 times, receiving nrTMS bursts at each of the six stimulation targets twice (20 Hz, 120% RMT and 140% RMT, respectively; 10s duration, ITI 40s). Stimulation parameters were selected in accordance with established SMA mapping protocols (Engelhardt et al., 2023; Kern et al., 2023) and remained within the device’s safety limits. For three participants, stimulation intensity was reduced for the LEMOCOT to avoid exceeding the stimulator’s maximum output. Due to technical issues, kinematic tracking failed for one participant during the LEMOCOT and for another during the NHPT.

Following six stimulation trains, participants were given at least 30 s of rest before completing the remaining trials. The order of stimulation targets was randomized for each participant and repeated across the experimental halves to ensure two stimulations per site.

The validation condition concluded testing for each modality, replicating the experimental procedure and stimulation parameters but targeting control sites outside of the SMA and M1. The stimulation of these 12 locations was randomized.

### 2.4 Data analysis

Movements were recorded across all sessions at Full-HD resolution and 100–120 FPS using a high-speed camera (GoPro, San Mateo, USA). Trial videos were standardized using the TMS device’s timing data, including a 2-s buffer to ensure complete task capture. For FACE trials, non-task-related facial movements (e.g., talking) were manually excluded.

For the NHPT and LEMOCOT, task performance was quantified using two complementary methods:

First, fine-grained kinematic data for each movement phase were recorded for each trial. Upper extremity and lower extremity movements were analyzed using Google MediaPipe Hands (GMH; Zhang et al., 2020), a deep learning framework that extracts 21 three-dimensional landmarks per frame from standardized video recordings.

For upper extremity tasks, landmark trajectories were normalized and segmented into discrete movement phases: peg pickup, travel, placement, and return. The duration of each phase, as well as the individual full movement duration (i.e., from pickup to return for each peg), was extracted. For lower extremity tasks, only the individual full movement duration was computed due to the absence of discrete phases. This approach enabled phase-specific assessment of motor performance beyond global task metrics. Further details on preprocessing, segmentation logic, and implementation are provided in the Supplementary Figures S1–S3.

Orofacial movements were analyzed using a series of pre-trained convolutional neural networks. BlazeFace (Bazarevsky et al., 2019) was used to detect facial bounding, followed by FaceMesh V2 (Yan and Grishchenko, 2022), which extracted 478 three-dimensional landmarks per frame. From these, blendshape estimates of facial muscle activation were computed using Blendshapes GHUM (Grishchenko et al., 2023). These blendshape values represent unitless, normalized values indicating the degree to which a specific facial expression or deformation is expressed. For each trial, mean blendshape intensity changes were calculated. Detailed information on processing steps is provided in the Supplementary Figures S4, S5.

Second, a coarse behavioral measure, the average time per peg placement in the NHPT or taps per second in the LEMOCOT, was included to validate the kinematic analysis. These measures were calculated by dividing the trial duration by the number of peg placements or taps, thereby reflecting the average movement duration of each placement or tap. No behavioral outcome was assessed for FACE trials.

### 2.5 Statistical analysis

Upper extremity, lower extremity, and orofacial motor tasks were analyzed separately using generalized estimating equations (GEEs), with participant ID included as a clustering variable and an exchangeable correlation structure. A Gaussian error distribution with an identity link was specified. Separate GEE models were fit for each outcome variable and modality, with stimulation condition (baseline, experimental, and validation) and stimulation site (anterior, middle, and posterior; within the experimental condition) included as categorical predictors.

For the upper extremities, models included average time per peg placement; individual durations of peg pickup, placement, and travel; and individual full movement durations as outcomes. For the lower extremities, taps per second and individual full movement durations were analyzed. Orofacial movements were modeled separately for each facial expression using the same GEE specifications. Prior to modeling, extreme values were winsorized at the 1st and 99th percentiles to mitigate tracking artifacts.

For all predictors, estimated marginal means were computed. *Post hoc* pairwise contrasts between factor levels were performed, and *p*-values were adjusted using the Benjamini–Hochberg procedure to control the false discovery rate. All testing was conducted at a significance level of *α* = 0.05.

All analyses and visualization were conducted in RStudio version 2024.12.1.563 (Posit Team, 2025) using R version 4.2 (R Core Team, 2024). All packages used and all statistical results are available in the Supplementary Tables S1–S13.

## 3 Results

### 3.1 Mapping of the upper extremity

**Figure 3:**
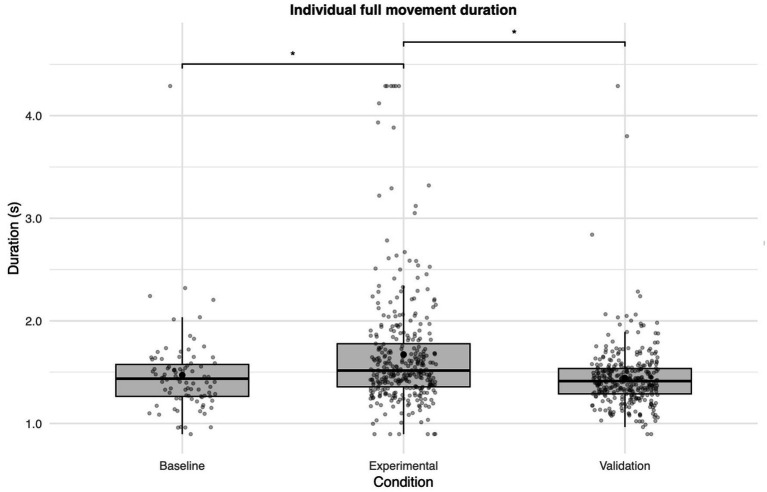
Effects of stimulation conditions on the individual full movement duration. Duration per full movement is shown for each condition. All *p*-values are labeled as *p* < 0.05 = *; *p*-values were adjusted using the Benjamini–Hochberg procedure for multiple comparisons.

The experimental condition had a significant effect on the individual full movement duration (*p* < 0.001, Figure 3). Post hoc tests showed longer durations in the experimental condition compared to both baseline and validation (both *p* < 0.001), with no difference between baseline and validation (*p* = 0.522). This pattern of results was reflected in the behavioral measure of time per peg placement, supporting the kinematic analysis (Supplementary Tables S1, S2).

**Figure 4:**
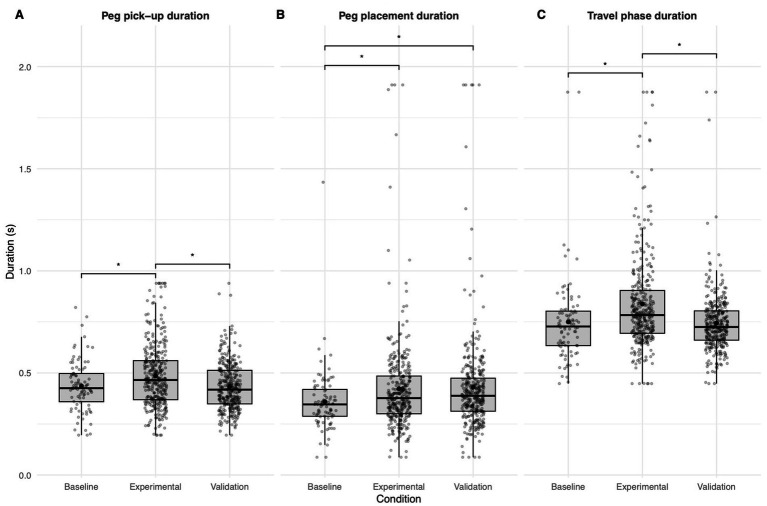
Phase-specific modulation of movement durations by stimulation condition. **(A)** Duration of the peg pick-up phase for each condition. **(B)** Duration of the peg placement phase for each condition. **(C)** Duration of the travel phase for each condition. All *p*-values are labeled as *p* < 0.05 = *; *p*-values were adjusted using the Benjamini–Hochberg procedure for multiple comparisons.

To identify which movement phases contributed to the overall slowing, phase-specific models were computed (Figure 4). All phases showed a significant effect of the experimental condition: peg pickup (*p* < 0.001), peg placement (*p* = 0.002), and travel duration (*p* < 0.001). Post hoc tests revealed that peg pickup and travel duration were significantly longer in the experimental condition compared to both baseline and validation (all *p* ≤ 0.001), with no difference between baseline and validation (pickup: *p* = 0.681; travel: *p* = 0.873). For peg placement, both the experimental (*p* = 0.006) and validation (*p* = 0.008) conditions differed from the baseline condition, but not from each other (*p* = 0.727).

To quantify contributions to the overall movement slowing, phase-wise increases relative to baseline were compared: pickup +0.049 s (11.2%), placement +0.064 s (17.8%), and travel +0.089 s (11.9%). While travel exhibited the largest absolute increase, placement showed the greatest relative change.

All stimulation sites significantly affected the individual full movement duration (all *p* < 0.030), with longer durations at middle (*p* = 0.047) and posterior (*p* = 0.043) sites compared to the anterior site, but no difference between middle and posterior sites (*p* = 0.692) in *post hoc* tests. Similar results were observed in the behavioral measure of time per peg placement, supporting the validity of the kinematic tracking (Supplementary Tables S3, S4).

Among movement phases, only travel duration was significantly modulated by stimulation site, showing increased durations at the posterior location (*p* = 0.019). Other phase models did not reach significance (all *p* ≥ 0.051), although the overall trend mirrored the behavioral data, with the strongest effects at posterior sites.

### 3.2 Mapping of the lower extremity

The individual full movement duration in the LEMOCOT was significantly reduced in both the experimental and validation conditions compared to baseline (both *p* < 0.001), with *post hoc* comparisons showing a reduction relative to baseline in both cases (both *p* < 0.001) and reduced duration for the validation condition compared to the experimental condition (*p* = 0.003).

This graded pattern, with fastest tapping in the validation condition compared to both the experimental and baseline conditions, was mirrored in taps per second, supporting the kinematic findings (Supplementary Tables S5, S6).

In the GEE model, no significant site effects were found for the individual full movement duration (all *p* ≥ 0.37), and all *post hoc* comparisons were non-significant (all *p* ≥ 0.489). This absence of significant effects was confirmed in the taps-per-second measure, with all post hoc comparisons remaining non-significant after correction for multiple comparisons (Supplementary Tables S7, S8).

### 3.3 Mapping of orofacial movements

Within GEE models for mean changes in blendshape, the validation condition significantly reduced activation for all facial movements except smiling (all *p* < 0.033). However, post hoc comparisons were significant only for cheek puffing, forceful eye closure, and lip puckering, where activation was lower in the validation condition compared to both the experimental and baseline conditions (all *p* < 0.029), with no difference between the experimental and baseline conditions (all *p* > 0.128).

In models evaluating the effect of stimulation site on mean changes in blendshape activation over time, only cheek puffing and pulling down the mouth corners were significantly affected. In both cases, the middle site led to larger mean changes than the anterior site (*p* = 0.028 and *p* = 0.044), while posterior stimulation had no significant effect (all *p* ≥ 0.108). No other movements showed significant site effects (all *p* ≥ 0.12), and no post hoc comparisons survived correction (all *p* > 0.098).

## 4 Discussion

We hypothesized that nrTMS stimulation across the SMA would produce disruptions in task performance, with no comparable effects during stimulation of non-motor regions. We expected that, within the SMA, task performance disruptions would localize to the subregion corresponding to each extremity, consistent with the proposed SMA somatotopy.

The study produced several key findings. First, SMA stimulation selectively affected upper extremity function, producing a clear anterior-to-posterior gradient of disruption, with the strongest effects in posterior regions. By decomposing performance into distinct movement phases, we identified that peg placement exhibited the largest increase in duration under SMA stimulation, suggesting a specific disruption in the fine motor sequence coordination required for this movement phase. Second, stimulation across the SMA did not selectively disrupt lower extremity motor function. Instead, performance appeared enhanced, with tapping speed highest during validation stimulation, intermediate during SMA stimulation, and lowest at baseline. No stimulation gradient was observed across SMA subregions for lower extremity tasks after correction. Third, we observed limited modulation of orofacial movements by SMA stimulation. Only a subset of facial expressions showed significant changes over time, and these effects were primarily observed during stimulation outside the SMA. After correction for multiple comparisons, no consistent spatial pattern of orofacial movement disruption could be identified within the SMA.

Previous research has shown that SMA stimulation can disrupt upper extremity task performance. Specifically, stimulation of six predefined SMA targets, analogous to those used in our study, has been shown to increase completion times on multiple subcomponents of the Jebsen–Taylor Hand Function Test and the NHPT (Schramm et al., 2019, 2020). Similarly, another nrTMS protocol demonstrated that SMA stimulation increases NHPT completion time (Kern et al., 2023). Consistent with these earlier findings, our results showed that nrTMS across the SMA selectively disrupts upper extremity motor function. Notably, one prior study (Engelhardt et al., 2023) revealed that stimulation over the medial to posterior aspects of the SMA produced the strongest disruption in complex upper extremity motor tasks.

The observed effects of SMA stimulation on upper extremity performance may be attributable to successful disruption of SMA processing, consistent with the virtual lesion paradigm used in TMS mapping (Krieg et al., 2017). The SMA has been suggested to be associated with the domain general and movement sequence processing (Cona and Semenza, 2017; Nachev et al., 2008), a task demand strongly represented in the NHPT. SMA disruption could therefore account for reduced NHPT performance, particularly reflected in the increased time required for peg placements, which demand fine motor sequence coordination.

Alternatively, the observed effects during posterior SMA stimulation on upper extremity tasks may, in part, reflect a methodological limitation related to unintended M1 co-activation. The observed pattern contrasts with previous findings indicating the middle SMA as most involved in upper extremity control (Fontaine et al., 2002; Krainik et al., 2004). This discrepancy may stem from the anatomical proximity of the posterior SMA to M1. High-intensity stimulation has been shown to activate non-primary motor areas through the activation of M1 (Mirbagheri et al., 2019), and field modeling studies have demonstrated that TMS can produce broad electrical fields at high intensities (Deng et al., 2012). As posterior SMA lies closest to M1, this region may be susceptible to overlapping activation.

Another neurobiologically plausible explanation is that the absence of a clear somatotopic gradient and the selective disruption limited to the upper extremity task reflect a task-dependent, rather than strictly effector-based, organization of the SMA. Prior research has shown that the SMA may not adhere to a strict effector-specific anatomical organization but instead exhibits a more flexible organization dependent on task demands and complexity (Rijntjes et al., 1999). Rijntjes et al. (1999) demonstrated that complex, overlearned movements can be represented in the same cortical location within the SMA despite being executed by different effectors, suggesting that the SMA may encode motor programs rather than specific effectors.

In line with this, Hiroshima et al. (2014) proposed the existence of a posterior “SMA core” that is consistently engaged across a variety of motor and language tasks, indicating a multifunctional zone that challenges the classical hypothesis of strict SMA somatotopy. These findings support a model of the SMA as a task-sensitive, functionally organized region, potentially explaining why only the upper extremity movement task was clearly disrupted in the present study and why effects were predominantly observed in the posterior SMA.

To account for the observation that changes in orofacial and lower extremity performance were greatest during the validation condition, despite stimulation being applied outside both M1 and the SMA and always tested last, it is important to consider methodological limitations such as the potential influence of peripheral and practice-related effects. For the lower extremities, performance improvements observed during the validation condition may reflect practice-related effects. Although such effects have been reported for the LEMOCOT in stroke patients (Menezes et al., 2017), they were modest and may not generalize to healthy individuals. Given that the validation phase was always performed last, practice-related effects may be a plausible explanation for the observed performance gains; under such circumstances, improved performance across successive runs within each condition would be expected. In the orofacial domain, significant changes were observed primarily under the non-motor validation condition. This effect may be attributable to peripheral stimulation, as the targeted sites were located near cutaneous facial muscles (Schumann et al., 2021). Given that TMS can directly activate superficial nerves and muscle fibers (Meteyard and Holmes, 2018), and that mapping facial muscles is technically challenging due to peripheral nerve stimulation artifacts and large motor thresholds (Säisänen et al., 2015), the observed changes in facial blendshape activation may result from peripheral, rather than cortical, effects. Alternatively, fatigue effects may have contributed to reduced activation under the validation condition, as the orofacial validation task was consistently performed last. Another limitation that may explain why disruptions were not strongest during SMA stimulation is that the task and its blendshape-based quantification have not yet been formally validated for sensitivity to SMA-related facial motor effects. Finally, the use of composite facial expression measures, such as blendshape, which quantify overall expression magnitude, may lack the sensitivity to detect effector-specific contributions of the SMA.

We were unable to identify a clear somatotopic organization within the SMA, which may be attributable to unintended M1 co-activation and the use of a predefined grid of six stimulation sites or the SMA’s role in higher-order motor planning rather than strict effector-specific somatotopy. Prior studies investigating SMA somatotopy have similarly failed to demonstrate the proposed anterior-to-posterior gradient corresponding to facial, upper extremity, and lower extremity representations (Schramm et al., 2019), and some did not systematically assess all effectors across all SMA subregions (e.g. Kern et al., 2023). None of these studies included a validation condition to assess stimulation specificity. Furthermore, substantial interindividual variability in the size and location of stimulation-responsive SMA regions has been reported (Kern et al., 2023; Engelhardt et al., 2023), possibly due to functional–anatomical differences or methodological factors such as coil orientation and targeting precision. Therefore, the absence of a consistent somatotopic pattern in our findings may reflect methodological limitations or the possibility that the SMA is organized according to functional rather than effector-specific somatotopy.

## 5 Conclusion

This study builds on prior nrTMS-based SMA mapping by systematically assessing its role in upper extremity, lower extremity, and orofacial motor control. We found that SMA stimulation selectively disrupted upper extremity performance, especially fine motor sequencing during peg placement, while no consistent effects emerged for lower extremity or orofacial movements. The absence of a clear somatotopic gradient may reflect methodological limitations or suggest that the SMA is organized functionally rather than according to effector-specific somatotopy. Importantly, we introduce a novel computer-vision-based method for phase-specific kinematic analysis, enabling more precise motor assessment. Future research should employ individualized targeting and M1 field modeling to further refine SMA topography.

## Data Availability

The raw data supporting the conclusions of this article can be made available upon reasonable request to the corresponding author.
