# Supplementary material for: Functional mapping of the somatotopic organization of the supplementary motor area using navigated repetitive transcranial magnetic stimulation and computer vision-based analysis
Source: Front Neurosci. 2026 Jan 30;20:1698148. doi: 10.3389/fnins.2026.1698148 (PMC12903118; doi:10.3389/fnins.2026.1698148)
Supplement: Supplementary file 1 [file Data_Sheet_1.docx]

Supplementary Material

## Supplementary Figures


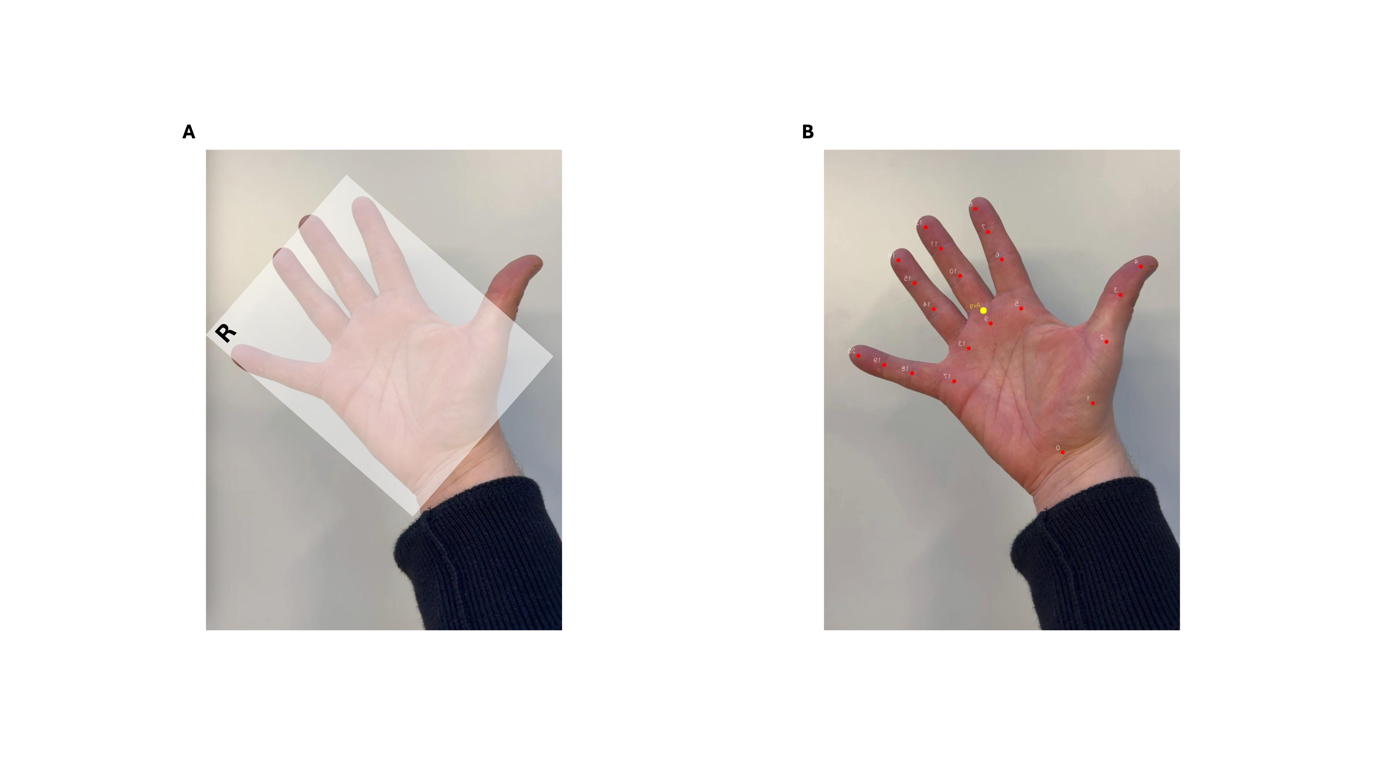
**Figure S1.** Acquisition of kinematic data of the upper and lower extremities: (A) BlazePalm Detector (Zhang et al., 2020) is employed to locate the palm of the hand within the frame (white box) and determine handedness. (B) Hand landmarks are then identified in the detected hand (red dots) and a average value (yellow dot) is computed using Google MediaPipe Hands (ibid.).

**
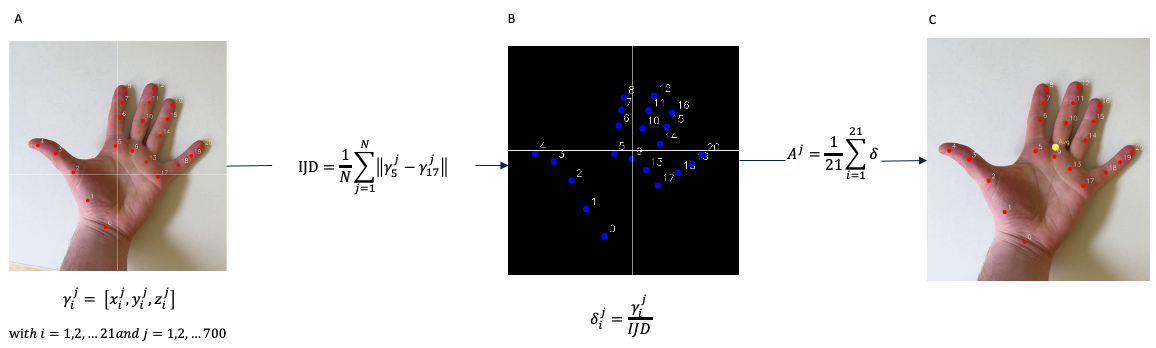
**

**Figure S2.** Data preprocessing: (A) Raw model data with landmarks across frames, (B) Inter-joint distance (IJD) as the Euclidean distance between landmarks 5 and 17, (C) Limb position as the average location of all landmarks. In this figure, $\gamma$ represents a three-dimensional coordinate with $x, y$ and $z$ coordinates. Indices $i$ denote the landmark, while indices $j$ represent the frame number. Additionally, $\delta_{i}^{j}$ indicates the normalized landmarks, and $A^{j}$ represents the normalized and averaged landmark.

**
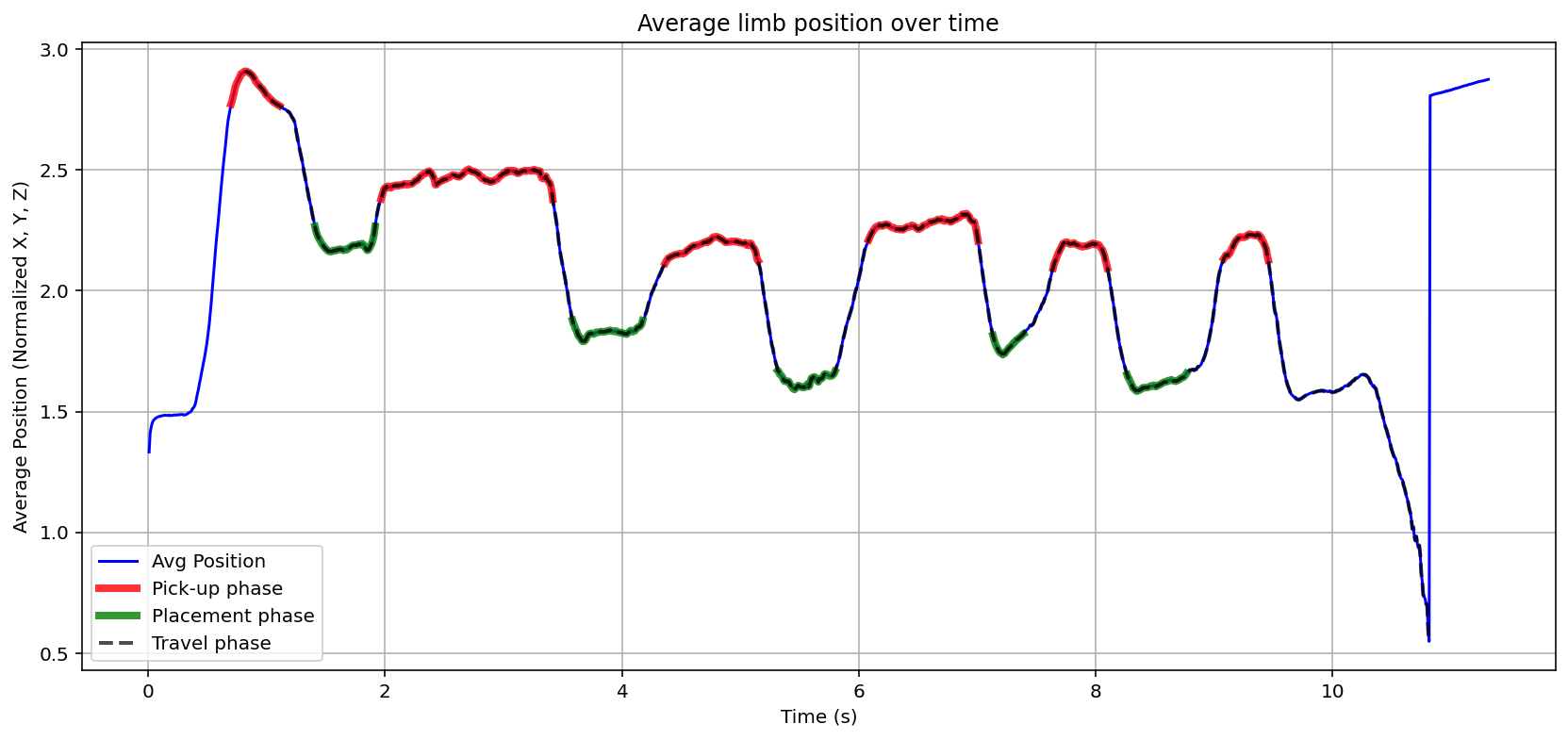
**

**Figure S3.** Example data for a single trial of the NHPT: Preprocessed data (i.e. normalized and scaled average hand position) is plotted over time for a single trial. Using SciPy (Virtanen et al., 2020), indices were identified where the average hand position exceeded its immediate neighbors with a prominence ≥0.2, capturing local maxima (peg placement) and minima (peg pick-up). These extrema defined plateau boundaries in the movement trajectory, with plateaus operationalized as segments where the signal remained within ±5% of the local extremum. Travel phases were defined as the intermediate segments. Duration off all segments were recorded. All trials were manually reviewed to ensure accurate phase segmentation and to validate automated detection.


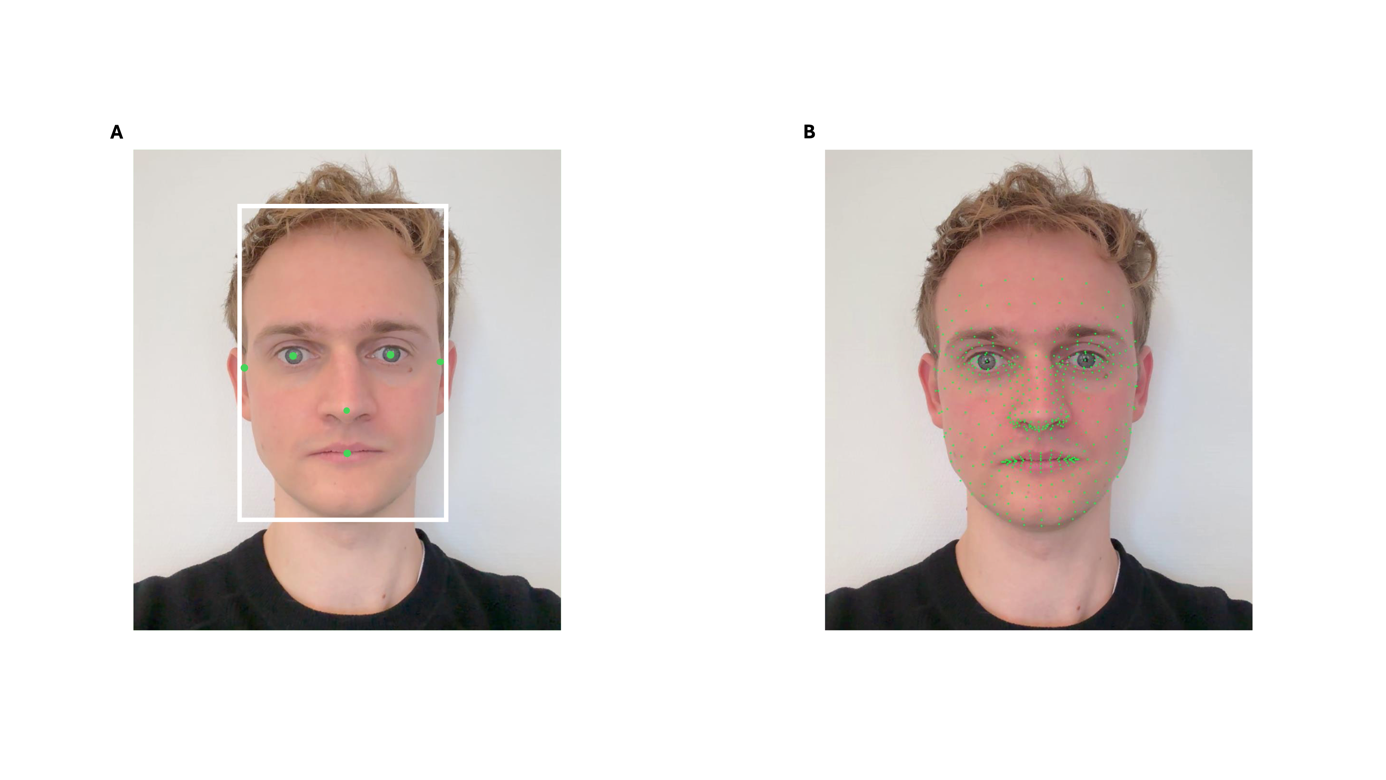


**Figure S4.** A sequence of pre-trained convolutional neural networks (CNNs) was used to detect and analyze facial features in each video frame. (A) BlazeFace (Bazarevsky et al., 2019) identified the facial bounding box and six core facial landmarks (green dots). (B) The cropped region was then passed to FaceMesh V2 (Yan & Grishchenko, 2022), yielding 478 3D facial landmarks (green dots). A subset of these was input to the Blendshape model (Grishchenko et al., 2023) after centering and normalization to estimate the intensity of individual facial muscle activations.


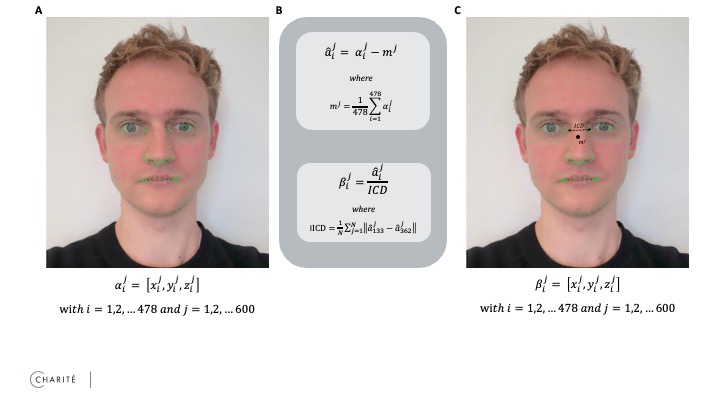


**Figure S5.** Facial movement preprocessing pipeline. (A) Raw facial landmark data extracted across frames. (B) Landmarks were centered relative to the face and spatially normalized. (C) Final output shows landmarks scaled by intercanthal distance (ICD) to account for individual facial size variability. In this figure, $\alpha$ represents a three-dimensional coordinate with $x, y$ and $z$values. Indices $i$ denote the landmark, while indices $j$ represent the frame number. Centered landmarks are denoted as $\hat{a}_{i}^{j}$ and normalized, centered landmarks are marked as $\beta_{i}^{j}$. The center of mass is denoted as $m$.

**List of References Pertaining to Supplementary Materials – Data Sheet 1**

Bazarevsky, V., Kartynnik, Y., Vakunov, A., Raveendran, K., & Grundmann, M. (2019). BlazeFace: Sub-millisecond Neural face Detection on mobile GPUs. *arXiv (Cornell University)*. https://doi.org/10.48550/arxiv.1907.05047

Grishchenko, I., Yan, G., Bazavan, E. G., Zanfir, A., Chinaev, N., Raveendran, K., Grundmann, M., & Sminchisescu, C. (2023). Blendshapes GHUM: Real-time Monocular facial blendshape prediction. *arXiv*. https://doi.org/10.48550/arxiv.2309.05782

Virtanen, P., Gommers, R., Oliphant, T. E., Haberland, M., Reddy, T., Cournapeau, D., Burovski, E., Peterson, P., Weckesser, W., Bright, J., Van Der Walt, S. J., Brett, M., Wilson, J., Millman, K. J., Mayorov, N., Nelson, A. R. J., Jones, E., Kern, R., Larson, E., . . . Vázquez-Baeza, Y. (2020). SciPy 1.0: fundamental algorithms for scientific computing in Python. *Nature Methods*, *17*(3), 261–272. https://doi.org/10.1038/s41592-019-0686-2

Yan, G., & Grishchenko, I. (2022). Model Card MediaPipe FaceMesh. In *Google AI for Developers*. https://storage.googleapis.com/mediapipe-assets/Model%20Card%20MediaPipe%20Face%20Mesh%20V2.pdf

Zhang, F., Bazarevsky, V., Vakunov, A., Tkachenka, A., Sung, G., Chang, C., & Grundmann, M. (2020). MediaPipe Hands: On-device real-time hand tracking. *arXiv (Cornell University)*. https://doi.org/10.48550/arxiv.2006.10214
