# Supplementary material for: Functional mapping of the somatotopic organization of the supplementary motor area using navigated repetitive transcranial magnetic stimulation and computer vision-based analysis
Source: Front Neurosci. 2026 Jan 30;20:1698148. doi: 10.3389/fnins.2026.1698148 (PMC12903118; doi:10.3389/fnins.2026.1698148)
Supplement: Supplementary file 2 [file Data_Sheet_2.docx]

Supplementary Material – Tables

**Supplementary Table 1**

*GEE Model Results for the upper extremity by condition*

| *Time per peg placement* | | | | |
| --- | --- | --- | --- | --- |
| Predictor | Estimate | Std. Error | Wald χ² | p-value |
| (Intercept) | 1.3958767 | 0.0503523 | 768.52 | <.001* |
| Condition: Experimental | 0.7081155 | 0.1970402 | 12.91 | <.001* |
| Condition: Validation | 0.0008073 | 0.0730776 | 0.00 | .991 |
| *Individual Full Movement Duration* | | | | |
| Predictor | Estimate | Std. Error | Wald χ² | p-value |
| (Intercept) | 1.4752 | 0.0530 | 773.62 | <.001* |
| Condition: Experimental | 0.1995 | 0.0463 | 18.57 | <.001* |
| Condition: Validation | -0.0284 | 0.0443 | 0.41 | .520 |
| *Peg pick-up duration* | | | | |
| Predictor | Estimate | Std. Error | Wald χ² | p-value |
| (Intercept) | 0.43605 | 0.0202 | 467.45 | <.001* |
| Condition: Experimental | 0.04873 | 0.0139 | 12.25 | <.001* |
| Condition: Validation | -0.00416 | 0.0101 | 0.17 | .681 |
| *Peg placement duration* | | | | |
| Predictor | Estimate | Std. Error | Wald χ² | p-value |
| (Intercept) | 0.3601 | 0.0165 | 474.72 | <.001* |
| Condition: Experimental | 0.0644 | 0.0208 | 9.59 | .002* |
| Condition: Validation | 0.0706 | 0.0251 | 7.88 | .005* |
| *Travel duration* | | | | |
| Predictor | Estimate | Std. Error | Wald χ² | p-value |
| (Intercept) | 0.75005 | 0.03086 | 590.74 | <.001* |
| Condition: Experimental | 0.08935 | 0.02454 | 13.25 | <.001* |
| Condition: Validation | -0.00476 | 0.02978 | 0.03 | .873 |

*Note.* p < .05 = *

**Supplementary Table 2**

*Post-Hoc Comparisons for the upper extremity by condition*

| *Time per peg placement* | | | | | | |
| --- | --- | --- | --- | --- | --- | --- |
| Contrast | Estimate | | SE | df | t-ratio | p-value |
| Baseline – Experimental | -0.7081 | | 0.1970 | 740 | -3.594 | .001* |
| Baseline – Validation | -0.0008 | | 0.0731 | 740 | -0.011 | .991 |
| Experimental – Validation | 0.7073 | | 0.2110 | 740 | 3.348 | .001* |
| *Individual Full Movement Duration* | |  |  |  |  |  |
| Contrast | Estimate | | SE | df | t-ratio | p-value |
| Baseline – Experimental | -0.1995 | | 0.0463 | 750 | -4.310 | <.001* |
| Baseline – Validation | 0.0284 | | 0.0443 | 750 | 0.640 | .522 |
| Experimental – Validation | 0.2279 | | 0.0504 | 750 | 4.520 | <.001* |
| *Peg pick-up duration* | | | | | | |
| Contrast | Estimate | | SE | df | t-ratio | p-value |
| Baseline – Experimental | -0.0487 | | 0.0139 | 752 | -3.500 | .001* |
| Baseline – Validation | 0.0042 | | 0.0101 | 752 | 0.410 | .681 |
| Experimental – Validation | 0.0529 | | 0.0127 | 752 | 4.170 | <.001* |
| *Peg placement duration* | | | | | | |
| Contrast | Estimate | | SE | df | t-ratio | p-value |
| Baseline – Experimental | -0.0644 | | 0.0208 | 752 | -3.097 | .006* |
| Baseline – Validation | -0.0706 | | 0.0252 | 752 | -2.807 | .008* |
| Experimental – Validation | -0.0062 | | 0.0178 | 752 | -0.350 | .727 |
| *Travel duration* | | | | | | |
| Contrast | Estimate | | SE | df | t-ratio | p-value |
| Baseline – Experimental | -0.0893 | | 0.0245 | 752 | -3.640 | <.001* |
| Baseline – Validation | 0.0048 | | 0.0298 | 752 | 0.160 | .873 |
| Experimental – Validation | 0.0941 | | 0.0215 | 752 | 4.380 | <.001* |

*Note.* p < .05 = *; p-values adjusted using the Benjamini–Hochberg correction for multiple comparisons

**Supplementary Table 3**

*GEE Model Results for the upper extremity by stimulation site*

| *Time per peg placement* | | | | |
| --- | --- | --- | --- | --- |
| Predictor | Estimate | Std. Error | Wald χ² | p-value |
| (Intercept) | 1.5138 | 0.0882 | 294.35 | <.001* |
| Middle Site | 0.5230 | 0.1981 | 6.97 | .008* |
| Posterior Site | 1.1803 | 0.2787 | 17.94 | <.001* |
| *Individual Full Movement Duration* | | | | |
| Predictor | Estimate | Std. Error | Wald χ² | p-value |
| (Intercept) | 1.5516 | 0.0512 | 919.52 | <.001* |
| Middle Site | 0.1661 | 0.0767 | 4.69 | .030* |
| Posterior Site | 0.2110 | 0.0858 | 6.05 | .014* |
| *Peg pick-up duration* | | | | |
| Predictor | Estimate | Std. Error | Wald χ² | p-value |
| (Intercept) | 0.47181 | 0.02124 | 493.61 | <.001* |
| Middle Site | 0.00278 | 0.01659 | 0.03 | .867 |
| Posterior Site | 0.03543 | 0.02040 | 3.02 | .082 |
| *Peg placement duration* | | | | |
| Predictor | Estimate | Std. Error | Wald χ² | p-value |
| (Intercept) | 0.3967 | 0.0218 | 331.25 | <.001* |
| Middle Site | 0.0497 | 0.0333 | 2.23 | .140 |
| Posterior Site | 0.0309 | 0.0188 | 2.71 | .100 |
| *Travel duration* | | | | |
| Predictor | Estimate | Std. Error | Wald χ² | p-value |
| (Intercept) | 0.7878 | 0.0218 | 1308.34 | <.001* |
| Middle Site | 0.0637 | 0.0327 | 3.80 | .051 |
| Posterior Site | 0.0918 | 0.0391 | 5.51 | .019* |

*Note.* p < .05 = *

**Supplementary Table 4**

*Post-Hoc Comparisons for the upper extremity by stimulation site*

| *Time per peg placement* | | | | | | |
| --- | --- | --- | --- | --- | --- | --- |
| Contrast | Estimate | | SE | df | t-ratio | p-value |
| Anterior– Middle | -0.523 | | 0.198 | 323 | -2.640 | .009* |
| Anterior– Posterior | -1.180 | | 0.279 | 323 | -4.240 | <.001* |
| Middle– Posterior | -0.657 | | 0.212 | 323 | -3.100 | .003* |
| *Individual Full Movement Duration* | |  |  |  |  |  |
| Contrast | Estimate | | SE | df | t-ratio | p-value |
| Anterior– Middle | -0.1661 | | 0.0767 | 334 | -2.165 | .047* |
| Anterior– Posterior | -0.2110 | | 0.0858 | 334 | -2.460 | .043* |
| Middle– Posterior | -0.0448 | | 0.1130 | 334 | -0.396 | .692 |
| *Peg pick-up duration* | | | | | | |
| Contrast | Estimate | | SE | df | t-ratio | p-value |
| Anterior– Middle | -0.0028 | | 0.0166 | 335 | -0.168 | .867 |
| Anterior– Posterior | -0.0354 | | 0.0204 | 335 | -1.737 | .125 |
| Middle– Posterior | -0.0326 | | 0.0161 | 335 | -2.030 | .125 |
| *Peg placement duration* | | | | | | |
| Contrast | Estimate | | SE | df | t-ratio | p-value |
| Anterior– Middle | -0.0497 | | 0.0333 | 335 | -1.493 | .205 |
| Anterior– Posterior | -0.0309 | | 0.0188 | 335 | -1.646 | .205 |
| Middle– Posterior | 0.0187 | | 0.0360 | 335 | 0.520 | .603 |
| *Travel duration* | | | | | | |
| Contrast | Estimate | | SE | df | t-ratio | p-value |
| Anterior– Middle | -0.0637 | | 0.0327 | 335 | -1.949 | .078 |
| Anterior– Posterior | -0.0918 | | 0.0391 | 335 | -2.348 | .058 |
| Middle– Posterior | -0.0281 | | 0.0466 | 335 | -0.603 | .547 |

*Note.* p < .05 = *; p-values adjusted using the Benjamini–Hochberg correction for multiple comparisons

**Supplementary Table 5**

*GEE Model Results for the lower extremity by condition*

| *Taps per second* | | | | |
| --- | --- | --- | --- | --- |
| Predictor | Estimate | Std. Error | Wald χ² | p-value |
| (Intercept) | 1.91727 | 0.10511 | 332.70 | <.001* |
| Condition: Experimental | 0.22216 | 0.04406 | 25.42 | <.001* |
| Condition: Validation | 0.36789 | 0.05206 | 49.93 | <.001* |
| *Individual Full Movement Duration* | | | | |
| Predictor | Estimate | Std. Error | Wald χ² | p-value |
| (Intercept) | 1.0599 | 0.0607 | 305.1 | <.001* |
| Condition: Experimental | -0.1155 | 0.0286 | 16.3 | <.001* |
| Condition: Validation | -0.1665 | 0.0261 | 40.8 | <.001* |

*Note.* p < .05 = *

**Supplementary Table 6**

*Post-Hoc Comparisons for the lower extremity by condition*

| *Taps per second* | | | | | | |
| --- | --- | --- | --- | --- | --- | --- |
| Contrast | Estimate | | SE | df | t-ratio | p-value |
| Baseline – Experimental | -0.222 | | 0.0441 | 764 | -5.042 | <.001* |
| Baseline – Validation | -0.368 | | 0.0521 | 764 | -7.066 | <.001* |
| Experimental – Validation | -0.146 | | 0.0235 | 764 | -6.201 | <.001* |
| *Individual Full Movement Duration* | |  |  |  |  |  |
| Contrast | Estimate | | SE | df | t-ratio | p-value |
| Baseline – Experimental | 0.116 | | 0.0286 | 753 | 4.040 | <.001* |
| Baseline – Validation | 0.167 | | 0.0260 | 753 | 6.390 | <.001* |
| Experimental – Validation | 0.051 | | 0.0174 | 753 | 2.930 | .003* |

*Note.* p < .05 = *; p-values adjusted using the Benjamini–Hochberg correction for multiple comparisons

**Supplementary Table 7**

*GEE Model Results for the lower extremity by stimulation site*

| *Taps per second* | | | | |
| --- | --- | --- | --- | --- |
| Predictor | Estimate | Std. Error | Wald χ² | p-value |
| (Intercept) | 2.1727 | 0.1291 | 283.20 | <.001* |
| Middle Site | -0.0241 | 0.0249 | 0.93 | .334 |
| Posterior Site | -0.0750 | 0.0315 | 5.69 | .017* |
| *Individual Full Movement Duration* | | | | |
| Predictor | Estimate | Std. Error | Wald χ² | p-value |
| (Intercept) | 0.9624 | 0.0628 | 234.88 | <.001* |
| Middle Site | -0.0218 | 0.0259 | 0.71 | .400 |
| Posterior Site | -0.0323 | 0.0364 | 0.71 | .370 |

*Note.* p < .05 = *

**Supplementary Table 8**

*Post-Hoc Comparisons for the lower extremity by stimulation site*

| *Taps per second* | | | | | | |
| --- | --- | --- | --- | --- | --- | --- |
| Contrast | Estimate | | SE | df | t-ratio | p-value |
| Anterior Site - Middle Site | 0.0241 | | 0.0250 | 336 | 0.965 | .335 |
| Anterior Site - Posterior Site | 0.0750 | | 0.0315 | 336 | 2.384 | .053 |
| Middle Site - Posterior Site | 0.0509 | | 0.0271 | 336 | 1.880 | .092 |
| *Individual Full Movement Duration* | |  |  |  |  |  |
| Contrast | Estimate | | SE | df | t-ratio | p-value |
| Anterior Site - Middle Site | 0.0218 | | 0.0259 | 334 | 0.841 | .489 |
| Anterior Site - Posterior Site | 0.0323 | | 0.0364 | 334 | 0.887 | .489 |
| Middle Site - Posterior Site | 0.0105 | | 0.0152 | 334 | 0.693 | .489 |

*Note.* p < .05 = *; p-values adjusted using the Benjamini–Hochberg correction for multiple comparisons

**Supplementary Table 9**

*GEE Model Results: Mean Face Blendshape Change Over Time by Condition*

| Raising Eyebrows | | | | |
| --- | --- | --- | --- | --- |
| Predictor | Estimate | Std. Error | Wald χ² | p-value |
| (Intercept) | 0.004312 | 0.000338 | 162.99 | <.001* |
| Condition: Experimental | -0.000384 | 0.000379 | 1.02 | .312 |
| Condition: Validation | -0.000786 | 0.000367 | 4.57 | .033 * |
| Pucker Lips | | | | |
| Predictor | Estimate | Std. Error | Wald χ² | p-value |
| (Intercept) | 0.001888 | 0.000148 | 162.73 | <.001* |
| Condition: Experimental | -0.000143 | 0.000119 | 1.45 | . 228 |
| Condition: Validation | -0.000401 | 0.000109 | 13.56 | <.001* |
| Blow out cheeks |  |  |  |  |
| Predictor | Estimate | Std. Error | Wald χ² | p-value |
| (Intercept) | 0.002326 | 0.000290 | 64.54 | <.001* |
| Condition: Experimental | -0.000337 | 0.000220 | 2.34 | .126 |
| Condition: Validation | -0.000687 | 0.000218 | 9.89 | .002* |
| Pulling down the corners of the mouth | | | | |
| Predictor | Estimate | Std. Error | Wald χ² | p-value |
| (Intercept) | 0.000896 | 0.000153 | 34.38 | <.001* |
| Condition: Experimental | -0.0000891 | 0.0000777 | 1.32 | .251 |
| Condition: Validation | -0.000166 | 0.000078 | 4.53 | .033* |
| Closing and Opening Eyes | | | | |
| Predictor | Estimate | Std. Error | Wald χ² | p-value |
| (Intercept) | 0.003469 | 0.000198 | 307.1 | <.001* |
| Condition: Experimental | 0.000166 | 0.000213 | 0.6 | .437 |
| Condition: Validation | -0.000525 | 0.000222 | 5.6 | .018* |
| Forming a full smile | | | | |
| Predictor | Estimate | Std. Error | Wald χ² | p-value |
| (Intercept) | 0.00228 | 0.000169 | 182.35 | <.001* |
| Condition: Experimental | –0.0000555 | 0.000136 | 0.17 | .680 |
| Condition: Validation | –0.000159 | 0.000128 | 1.54 | .220 |

*Note.* p < .05 = *

**Supplementary Table 10**

*Post-Hoc Comparisons: Mean Face Blendshape Change Over Time by Condition*

| Raising Eyebrows | | | | | |
| --- | --- | --- | --- | --- | --- |
| Contrast | Estimate | SE | df | t-ratio | p-value |
| Baseline – Experimental | 0.000384 | 0.000379 | 139 | 1.011 | .314 |
| Baseline – Validation | 0.000786 | 0.000367 | 139 | 2.138 | .052 |
| Experimental – Validation | 0.000402 | 0.000188 | 139 | 2.137 | .052 |
| Pucker Lips |  |  |  |  |  |
| Contrast | Estimate | SE | df | t-ratio | p-value |
| Baseline – Experimental | 0.0000555 | 0.000136 | 143 | 1.210 | .230 |
| Baseline – Validation | 0.000159 | 0.000128 | 143 | 3.680 | .001* |
| Experimental – Validation | 0.000103 | 0.0000914 | 143 | 2.770 | .009* |
| Blow out cheeks | | | | | |
| Contrast | Estimate | SE | df | t-ratio | p-value |
| Baseline – Experimental | 0.000337 | 0.000220 | 143 | 1.530 | .128 |
| Baseline – Validation | 0.000687 | 0.000218 | 143 | 3.145 | .006* |
| Experimental – Validation | 0.000350 | 0.000122 | 143 | 2.861 | .007* |
| Pulling down the corners of the mouth | | | | | |
| Contrast | Estimate | SE | df | t-ratio | p-value |
| Baseline – Experimental | 0.0000891 | 0.0000777 | 141 | 1.147 | .253 |
| Baseline – Validation | 0.000166 | 0.0000780 | 141 | 2.128 | .105 |
| Experimental – Validation | 0.0000768 | 0.0000654 | 141 | 1.175 | .253 |
| Closing and Opening Eyes | | | | | |
| Contrast | Estimate | SE | df | t-ratio | p-value |
| Baseline – Experimental | -0.000166 | 0.000213 | 140 | -0.780 | .438 |
| Baseline – Validation | 0.000525 | 0.000222 | 140 | 2.370 | .029* |
| Experimental – Validation | 0.000691 | 0.000190 | 140 | 3.640 | .001* |
| Forming a full smile | | | | | |
| Contrast | Estimate | SE | df | t-ratio | p-value |
| Baseline – Experimental | 0.0000555 | 0.000136 | 143 | 0.407 | .685 |
| Baseline – Validation | 0.000159 | 0.000128 | 143 | 1.239 | .393 |
| Experimental – Validation | 0.000103 | 0.0000914 | 143 | 1.127 | .393 |

*Note.* p < .05 = *

**Supplementary Table 11**

*GEE Model Results: Mean Face Blendshape Change Over Time by Stimulation Site*

| Raising Eyebrows | | | | |
| --- | --- | --- | --- | --- |
| Predictor | Estimate | Std. Error | Wald χ² | p-value |
| (Intercept) | 0.004298 | 0.000507 | 71.95 | <.001* |
| Middle Site | -0.000341 | 0.000903 | 0.14 | .710 |
| Posterior Site | -0.000579 | 0.000583 | 0.98 | .320 |
| Pucker Lips | | | | |
| Predictor | Estimate | Std. Error | Wald χ² | p-value |
| (Intercept) | 0.001811 | 0.000277 | 42.86 | <.001* |
| Middle Site | 0.000099 | 0.000349 | 0.08 | .780 |
| Posterior Site | -0.000285 | 0.000312 | 0.84 | .360 |
| Blow out cheeks |  |  |  |  |
| Predictor | Estimate | Std. Error | Wald χ² | p-value |
| (Intercept) | 0.001401 | 0.000262 | 28.55 | <.001* |
| Middle Site | 0.001282 | 0.000585 | 4.80 | .028* |
| Posterior Site | 0.000696 | 0.000433 | 2.58 | .108 |
| Pulling down the corners of the mouth | | | | |
| Predictor | Estimate | Std. Error | Wald χ² | p-value |
| (Intercept) | 0.000775 | 0.000176 | 19.35 | <.001* |
| Middle Site | 0.000355 | 0.000176 | 4.06 | .044* |
| Posterior Site | 0.000109 | 0.000208 | 0.27 | .602 |
| Closing and Opening Eyes | | | | |
| Predictor | Estimate | Std. Error | Wald χ² | p-value |
| (Intercept) | 0.004542 | 0.000707 | 41.33 | <.001* |
| Middle Site | -0.000925 | 0.000729 | 1.61 | .200 |
| Posterior Site | -0.001201 | 0.000768 | 2.44 | .120 |
| Forming a full smile | | | | |
| Predictor | Estimate | Std. Error | Wald χ² | p-value |
| (Intercept) | 0.002265 | 0.000277 | 66.70 | <.001* |
| Middle Site | -0.000193 | 0.000277 | 0.34 | .560 |
| Posterior Site | 0.000457 | 0.000462 | 0.98 | .320 |

*Note.* p < .05 = *

**Supplementary Table 12**

*Post-Hoc Comparisons: Mean Face Blendshape Change Over Time by Stimulation Site*

| Raising Eyebrows | | | | | |
| --- | --- | --- | --- | --- | --- |
| Contrast | Estimate | SE | df | t-ratio | p-value |
| Anterior– Middle | 0.000341 | 0.000903 | 55 | 0.377 | .751 |
| Anterior – Posterior | 0.000579 | 0.000583 | 55 | 0.992 | .751 |
| Middle – Posterior | 0.000238 | 0.000747 | 55 | 0.319 | .751 |
| Pucker Lips |  |  |  |  |  |
| Contrast | Estimate | SE | df | t-ratio | p-value |
| Anterior– Middle | -0.000099 | 0.000349 | 55 | -0.283 | .778 |
| Anterior – Posterior | 0.000285 | 0.000312 | 55 | 0.914 | .547 |
| Middle – Posterior | 0.000384 | 0.000247 | 55 | 1.557 | .376 |
| Blow out cheeks | | | | | |
| Contrast | Estimate | SE | df | t-ratio | p-value |
| Anterior– Middle | -0.001282 | 0.000585 | 55 | -2.191 | .098 |
| Anterior – Posterior | -0.000696 | 0.000433 | 55 | -1.607 | .171 |
| Middle – Posterior | 0.000586 | 0.000512 | 55 | 1.145 | .257 |
| Pulling down the corners of the mouth | | | | | |
| Contrast | Estimate | SE | df | t-ratio | p-value |
| Anterior– Middle | -0.000355 | 0.000176 | 53 | -2.016 | .147 |
| Anterior – Posterior | -0.000109 | 0.000208 | 53 | -0.522 | .604 |
| Middle – Posterior | 0.000247 | 0.000154 | 53 | 1.606 | .171 |
| Closing and Opening Eyes | | | | | |
| Contrast | Estimate | SE | df | t-ratio | p-value |
| Anterior– Middle | 0.000925 | 0.000729 | 54 | 1.269 | .315 |
| Anterior – Posterior | 0.001201 | 0.000768 | 54 | 1.563 | .315 |
| Middle – Posterior | 0.000275 | 0.000484 | 54 | 0.569 | .571 |
| Forming a full smile | | | | | |
| Contrast | Estimate | SE | df | t-ratio | p-value |
| Anterior– Middle | 0.000193 | 0.000330 | 55 | 0.586 | .560 |
| Anterior – Posterior | -0.000457 | 0.000462 | 55 | -0.989 | .491 |
| Middle – Posterior | -0.000651 | 0.000406 | 55 | -1.601 | .345 |

*Note.* p < .05 = *

**Supplementary Table 13**

*List of software packages used in the analysis*

| Package | Citation |
| --- | --- |
| geepack | Halekoh, U., Højsgaard, S., & Yan, J. (2006). The R Package Geepack for generalized estimating equations. *Journal of Statistical Software*, *15*(2). https://doi.org/10.18637/jss.v015.i02 |
| emmeans | Lenth, R. (2025). *emmeans: Estimated Marginal Means, aka Least-Squares Means* (1.11.1) [Software]. https://CRAN.R-project.org/package=emmeans |
| dplyr | Wickham, H., François, R., Henry, L., Müller, K., & Vaughan, D. (2023). *dplyr: A Grammar of Data Manipulation* (1.1.4) [Software]. https://CRAN.R-project.org/package=dplyr |
| ggplot2 | Wickham, H. (2016). *ggplot2: Elegant Graphics for Data Analysis*. Springer. |
| DescTools | Signorell, A. (2025). *DescTools: Tools for Descriptive Statistics* (0.99.60) [Software]. https://CRAN.R-project.org/package=DescTools |
| readr | Wickham, H., Hester, J., & Bryan, J. (2024). *readr: Read Rectangular Text Data* (2.1.5) [Software]. https://CRAN.R-project.org/package=readr |
| readxl | Wickham, H., & Bryan, J. (2025). *readxl: Read Excel Files* (1.4.5) [Software]. https://CRAN.R-project.org/package=readxl |
| ggsignif | Ahlmann-Eltze, C., & Patil, I. (2021). ggsignif: R Package for Displaying Significance Brackets for “ggplot2.” *PsyArxiv*. https://doi.org/10.31234/osf.io/7awm6 |
| cowplot | Wilke, C. O. (2024). *cowplot: Streamlined Plot Theme and Plot Annotations for “ggplot2”* [Software]. https://CRAN.R-project.org/package=cowplot |
